# Séroprévalence des anticorps anti-virus de l´hépatite C et facteurs associés, d´après un dépistage volontaire en population générale en 2016 au Bénin

**DOI:** 10.11604/pamj.2021.40.30.28775

**Published:** 2021-09-10

**Authors:** Aboudou Raïmi Kpossou, Benoît Kouwakanou, Comlan N´déhougbèa Martin Sokpon, Khadidjatou Saké Alassane, Marc Moboladji Bankolé, Carin Ahouada, Rodolph Koffi Vignon, Vincent Zoundjiekpon, Fadel Sourokou, Jean Séhonou, Nicolas Kodjoh

**Affiliations:** 1 Clinique Universitaire d’Hépato-gastroentérologie, Centre National Hospitalier et Universitaire Hubert Koutoukou Maga (CNHU-HKM), Cotonou, Bénin,; 2 Service d´Hépato-gastroentérologie, CHU Mohammed VI de Marrakech, Marrakech, Maroc,; 3 Service de Médecine Interne, Centre Hospitalier Universitaire Départemental Borgou-Alibori, Parakou, Bénin,; 4 Unité de Formation et de Recherche en Pharmacie, Faculté des Sciences de la Santé, Université d´Abomey-Calavi, Cotonou, Bénin,; 5 Service de Médecine, Hôpital de Zone d´Allada, Allada, Bénin,; 6 Service d´Hépato-gastroentérologie et Gériatrie, Hôpital Universitaire d´Olomouc, Olomouc, République Tchèque,; 7 Clinique Internationale de Cotonou Aupiais, Cotonou, Bénin,; 8 Programme National de Lutte contre l´Hépatite, Ministère de la Santé, Cotonou, Bénin

**Keywords:** Virus de l’hépatite C, prévalence, facteurs associés, Bénin, Hepatitis C virus, prevalence, associated factors, Benin

## Abstract

**Introduction:**

l´hépatite C est un problème de santé publique dans le monde, et plus particulièrement en Afrique subsaharienne. L´objectif de ce travail était de déterminer la séroprévalence des anticorps anti-virus de l´hépatite C ainsi que les facteurs associés, à l´occasion d´un dépistage volontaire en population générale au Bénin.

**Méthodes:**

il s´agissait d´une étude transversale descriptive et analytique qui s´était déroulée en juillet 2016 dans 4 grandes villes de 4 différents départements du Bénin. Etaient inclus tous les volontaires résidents dans ces villes ciblées, de tous âges, ayant donné leur consentement éclairé. Pour cette recherche, le test de diagnostic rapide HCV ImuMed (Healgen Scientific LLC, USA) avait été utilisé. Une analyse par régression logistique avait été utilisée afin d´identifier les facteurs associés à l´infection par le virus de l´hépatite C.

**Résultats:**

au total, 2809 volontaires étaient inclus avec une moyenne d´âge de 25,9± 16,5 ans (les extrêmes: 0 et 86 ans), constitués de 53,9% (1514/2809) d´hommes et de 46,1% (1295/2809) de femmes. Plus de la moitié de la population d´étude était constituée de célibataires 59,1% (1612/2726). Il s´agissait principalement de 41,3% (1074/2809) d´élèves ou étudiants. La séroprévalence du VHC était de 1,5% (42/2809). En analyse multivariée, les variables significativement associées au portage des anti-VHC étaient: l´âge de 60 ans et plus (aOR: 46,9, IC 95% 10,2-216,0; p < 0,0001) et l´antécédent d´alcoolisme (aOR: 6,3, IC 95% 3,3-12,1; p < 0,0001).

**Conclusion:**

la séroprévalence des anticorps anti-VHC était de 1,5% dans la population étudiée. L´infection par le VHC touchait volontiers les sujets âgés (de 60 ans et plus) et ceux ayant un antécédent d´alcoolisme.

## Abstract (en)

**Introduction:**

hepatitis C is a public health problem worldwide, in particular in sub-Saharan Africa. The purpose of this study is to determine the seroprevalence of hepatitis C virus antibodies and associated factors during a voluntary general population screening program in Benin.

**Method:**

we conducted a descriptive and analytical cross-sectional study in 4 big cities of 4 different departments in Benin in July 2016. All volunteers of all ages, residing in these targeted cities, who gave their informed consent were included in the study. ImuMed HCV Rapid Diagnostic Test (Healgen Scientific LLC, USA) was used. Logistic regression analysis was also used to identify factors associated with hepatitis C virus infection.

**Results:**

a total of 2809 volunteers with an average age of 25.9 ± 16.5 years (ranging from 0 to 86 years) were included in the study; 53.9% (1514/2809) of them were men and 46.1% (1295/2809) were women. More than half of the study population consisted of single (59.1%; 1612/2726); 41.3% (1074/2809) were pupils or students. VHC Seroprevalence was 1.5% (42/2809). In multivariate analysis, the variables significantly associated with anti-HCV carriage were: be 60 years old and older (aOR: 46.9, 95% CI 10.2-216.0; p<0.0001) and a history of alcoholism (aOR: 6.3; 95% CI 95% 3.3-12.1; p < 0.0001).

**Conclusion:**

in the general population, the seroprevalence of anti-HCV antibodies was 1.5%. HCV infection mainly occurred in people aged 60 years and older and in those with a history of alcoholism.

## Introduction

L´hépatite C est une maladie inflammatoire du foie causée par le virus de l´hépatite C (VHC). Elle passe souvent inaperçue à la phase aiguë mais évolue dans la majorité des cas vers la chronicité avec le risque de développer des complications telles que la cirrhose et le carcinome hépatocellulaire (CHC) [1]. La principale voie de transmission du VHC est la voie sanguine, par des expositions percutanées directes importantes ou répétées: transfusion, consommation de drogues par voie injectable ou partage de matériel d´injection, ou utilisation de matériel souillé pour des actes entraînant une effraction cutanée (scarifications, tatouage, piercing, acupuncture, excision, circoncision non médicalisée) [1-5]. La prévalence mondiale de l´hépatite C est estimée à 1% avec une répartition variable dans les différentes régions du globe, parfois même au sein d´un même pays [2]. Les zones de fortes endémicités sont représentées par la région de la Méditerranée Orientale (prévalence de 2,3%) suivie de la région de l´Union Européenne (prévalence de 1,5%) [2, 3]. Le continent africain se situe dans les zones de moyenne endémicité (prévalence de 1% en 2015) [2] avec une zone de forte endémicité au Nord, notamment en Egypte [2].

Au Bénin, la prévalence de l´hépatite C dans la population générale n´est pas connue. Certaines études antérieures avaient objectivé des prévalences variables (4,12% à 20%) en fonction de la population d´étude [5-7]. Une autre étude menée dans la clinique universitaire d´Hépato-gastroentérologie du Centre National Hospitalier Universitaire Hubert Koutoukou Maga (CNHU - HKM) de Cotonou avait montré que l´hépatite C touche beaucoup plus les personnes du 3^e^ âge (plus de 60 ans) avec une proportion de 62,7% des cas et une légère prédominance féminine (54,2%) [8]. L´avènement des antiviraux d´action directe (AAD) a révolutionné la prise en charge de l´infection au VHC de par son efficacité, sa tolérance, permettant ainsi de diminuer la survenue des complications mais aussi certaines manifestations extra-hépatiques [2, 3, 9, 10]. Dès lors, l´Organisation Mondiale de la Santé (OMS) s´est fixé comme objectif l´élimination du VHC à l´horizon 2030. Pour y parvenir, il importe de disposer de données factuelles sur la maladie dans les différents pays. C´est dans ce cadre que nous avons réalisé ce travail, à l´occasion de la journée mondiale contre l´hépatite en 2016. L´objectif était de déterminer la séroprévalence des anticorps anti-VHC ainsi que les facteurs associés, lors d´un dépistage volontaire en population générale au Bénin.

## Méthodes

### Conception et contexte de l´étude

Il s´agissait d´une étude transversale descriptive et analytique qui s´était déroulée en juillet 2016 à l´occasion de la célébration de la journée mondiale contre les hépatites dans les quatre plus grandes villes de quatre différents départements du Bénin (Cotonou, Porto-Novo situés dans les départements du Littoral et de l´Ouémé au Sud du Bénin ; Parakou et Natitingou situés dans les départements du Borgou et de l´Atacora au Nord du Bénin). En effet, un dépistage volontaire de masse de l´hépatite C était organisé dans ces villes par la Société Béninoise d´Hépato-gastroentérologie avec l´appui financier et technique du groupe Lefalco (Cotonou, Bénin) et du laboratoire pharmaceutique Genix Pharma.

### Population d´étude

Il s´agit d´un dépistage volontaire de masse de l´hépatite C organisé dans les villes ci-dessus citées au cours de la journée mondiale de lutte contre les hépatites virales. Etaient inclus tous les volontaires résidents dans les villes ciblées, de tous âges et ayant donné leur consentement éclairé. En cas de test positif, le sujet était informé par l´agent de santé membre de l´équipe de dépistage. Il le rassurait de ce que, il devra être contacté par un membre de la Société Béninoise d´Hépato-gastroentérologie pour des tests de confirmation et une prise en charge tandis qu´en cas de test négatif, le patient était rassuré qu´il n´avait pas l´infection par le VHC. Les conseils appropriés sur la prévention de l´infection lui étaient donnés.

### Collecte des données

Il s´agit d´une collecte de données prospective au cours du dépistage. Une fiche d´exploitation a été élaborée et remplie au fur et à mesure au cours du dépistage. Cette fiche comporte des variables sociodémographiques et les antécédents. Un prélèvement de sang était réalisé pour la recherche d´anticorps anti-VHC. Pour cette recherche, le test de diagnostic rapide (TDR) HCV ImuMed (Healgen Scientific LLC, USA) avait été utilisé. La procédure de réalisation indiquée par le fabricant avait été suivie.

### Définitions des variables

La variable dépendante était l´anticorps anti-HVC détecté par TDR (positif ou négatif). Les variables indépendantes étaient: sociodémographiques (l´âge, le sexe, la profession, le niveau d´instruction, le lieu de résidence), antécédents (les antécédents de contact avec le sang notamment par transfusion ou scarification, ou de maladie non transmissible comme le diabète).

### Analyse statistique

Les données issues de cette opération de dépistage avaient permis de mettre en facteur les variables qu'étaient: l´âge, le sexe, la profession, le niveau d´instruction, le lieu de résidence, les antécédents de contact avec le sang (transfusion, scarification), la maladie non transmissible (le diabète). Les données avaient été saisies avec Excel. L´analyse statistique était faite dans SAS studio 9.4. Les variables continues avaient été exprimées sous forme de moyennes avec leur écart type ou de médianes avec leurs intervalles interquartiles, des variables catégorielles avaient été exprimées en pourcentages. Une analyse univariée par régression logistique simple avait permis d´identifier les facteurs sociodémographiques et antécédents associés à l´infection de l´hépatite C. La stratégie d´analyse multivariée avait consisté à inclure toutes les variables associées à l´infection de l´hépatite C au seuil p < 0,20. La procédure manuelle descendante avait été utilisée pour obtenir le modèle final. Les rapports de cotes ajustés et leurs intervalles de confiance à 95% avaient été calculés.

### Considérations éthiques

Le consentement verbal de chaque participant était obtenu avant son inclusion dans l´étude. La confidentialité des résultats du test de dépistage ainsi que des données recueillies auprès de chaque participant a été assurée durant tout le processus. L´étude n´a pas été soumise au comité local d´éthique.

## Résultats

### Caractéristiques de la population étudiée

Au total 2809 volontaires constitués de 53,90% de sexe masculin et de 46,1% de sexe féminin, soit un sex-ratio de 1,2, répartis sur les quatre grandes villes dans lesquelles avait eu lieu le dépistage. La répartition par ville des participants se présentait comme suit : 12,5% (352/2809) à Porto-Novo, 25,8% (725/2809) à Cotonou, 28,9% (811/2809) à Parakou et 32,8% (921/2809) à Natitingou. La moyenne d´âge des participants était de 25,9 ± 16,5 ans avec une médiane de 24 ans (les âges extrêmes étant 0 et 86 ans). Plus de la moitié de la population d´étude était constituée de célibataires 59,1% (1612/2726) et un peu plus du 1/3 était en couple 39,4% (1086/2726). Il s´agissait principalement d´élèves ou étudiants (1074/2809, 41,3%), de cadres moyens (214/2599, 8,2%), d´artisans/ménagères (203/2599, 7,8%) et d´ouvriers (187/2599, 7,2%) (Tableau 1).

**Tableau 1:** caractéristiques générales des participants au dépistage de VHC, Bénin 2016

	Effectif	%
Sexe (n= 2809)		
**Masculin**	1514	53,90
**Féminin**	1295	46,10
Groupes d´âge [en année] (n=2809)		
**Moins de 18**	1028	57,72
**[18-25[**	448	25,15
**[25-32[**	399	22,40
**[32-39[**	303	17,01
**[39-46[**	252	14,15
**[46-53[**	164	9,21
**[53-60[**	111	6,23
**60 et plus**	104	5,84
Statut Matrimonial (n= 2726)*		
**Célibataires**	1612	59,13
**En couple**	1086	39,84
**Divorcé(e) /Veuf (Veuve)**	28	1,03
Niveau d´étude (n=2411)*		
**Primaire**	719	29,82
**Secondaire**	933	38,70
**Supérieur**	550	22,81
**Illettré et autres**	209	8,67
Catégories professionnelles (n=2603)*		
**Ménagères/ Artisans**	203	7,80
**Ouvriers**	187	7,18
**Cadres supérieurs**	131	5,03
**Cadres moyens**	214	8,22
**Cadres inférieurs**	113	4,34
**Militaires**	76	2,92
**Elèves/ Etudiants**	1074	41,26
**Commerçants**	218	8,37
**Autres**	387	14,87
Répartition des cas dans les villes de résidence (n= 2809)		
**Porto - Novo**	352	12,53
**Cotonou**	725	25,81
**Parakou**	811	28,87
**Natitingou**	921	32,79
Antécédents		
Diabétique (n= 2809)		
**Oui**	82	2,92
**Non**	2725	97,08
Transfusion Sanguine (n=2809)		
**Oui**	118	4,20
**Non**	2691	95,80
Alcoolisme (n=2809)		
**Oui**	401	14,28
**Non**	2408	85,72
Hépatite B (n=2809)		
**Oui**	194	6,91
**Non**	2615	93,09
Hépatite C (n=2809)		
**Oui**	53	1,89
**Non**	2756	98,11
VIH (n=2809)		
**Oui**	223	7,94
**Non**	2586	92,06
Cirrhose (n=2809)		
**Oui**	105	3,74
**Non**	2704	96,26
(*) : Données manquantes		

### Séroprévalence des anticorps anti-VHC et facteurs associés

La séroprévalence globale de l´infection au virus de l´hépatite C dans l´ensemble de la population dépistée était de 1,5% (42 personnes testées positives sur 2809) [IC95% = 0,63% - 2,15%]. En analyse univariée, étaient associées à l´infection par le VHC, l´âge ]53 - 60 ans] et de ]60 ans et plus[, la vie en couple et un antécédent d´alcoolisme. En analyse multivariée, les variables qui étaient significativement associées au VHC sont: âge de 60 ans et plus: aOR: 46,9, IC95%: 10,2-216,0 et p < 0,0001; l´ATCD d´alcoolisme: aOR: 6,3, IC95%: 3,3-12,1 et p < 0,0001 (Tableau 2).

**Tableau 2:** analyse uni et multi variée de l´infection au virus de l´hépatite C en population générale selon les caractéristiques sociodémographiques et les antécédents, Bénin 2016

	Hépatite C n(%)	Analyse univariée			Analyse multi variée		
		OR	IC95%	p	AOR	IC95%	p
Sexe (n= 2809)							
**Masculin**	21(50)	1			-	-	-
**Féminin**	21(50)	1,2	0,6-2,2	0,61	-	-	-
Groupes déâge [en année] (n=2809)							
**Moins de 18**	2(4,8)	1			1		
**[18-25[**	5(11,9)	5,8	1,1-29,9	**0,03**	3,6	0,7-19,0	0,13
**[25-32[**	4(9,5)	5,2	0,9-28,5	**0,05**	2,7	0,5-15,1	0,27
**[32-39[**	4(9,5)	6,9	1,3-37,7	**0,02**	3,5	0,6-20,1	0,15
**[39-46[**	2(4,8)	4,1	0,6-29,3	0,15	2,3	0,3-16,7	0,41
**[46-53[**	4(9,5)	12,8	2,3-70,6	**0,003**	6,5	1,1-37,4	**0,03**
**[53-60[**	7(16,7)	34,5	7,1-168,0	**<0,0001**	21,0	4,2-105,5	**0,0002**
**60 et plus**	14(33,3)	79,8	17,9-356,6	**<0,0001**	46,9	10,2-216,0	**<0,0001**
Statut Matrimonial (n= 2726)*							
**Célibataires**	7(17,1)	1			-	-	-
**En couple**	32(78,1)	6,7	3,1-15,9	**<0,0001**	-	-	-
**Divorcé(e) /Veuf (Veuve)**	2(4,9)	17,6	3,5-89,0	**0,0005**	-	-	-
Niveau d´étude (n=2411)*							
**Primaire**	3(7,1)	1					
**Secondaire**	21(50)	5,5	1,6-18,5	**0,005**	-	-	-
**Supérieur**	17(40,3)	7,6	2,2-26,1	**0,001**	-	-	-
**Illettré et autres**	1(2,4)	1,1	0,1-11,1	0,90	-	-	-
Catégories professionnelles (n=2603)*							
**Ménagères/ Artisans**	4(9,5)	1					
**Ouvriers**	2(4,8)	0,5	0,1-3,0	0,47	-	-	-
**Cadres supérieurs**	5(11,9)	0,6	0,1-5,5	0,65	-	-	-
**Cadres moyens**	5(11,9)	0,3	0,0-2,7	0,28	-	-	-
**Cadres inférieurs**	5(11,9)	0,8	0,1-7,1	0,82	-	-	-
**Militaires**	3(7,1)	6,0	1,3-28,2	**0,02**	-	-	-
**Elèves/ Etudiants**	4(9,5)	0,2	0,1-0,8	**0,01**	-	-	-
**Commerçants**	3(7,1)	0,7	0,1-3,1	0,63	-	-	-
**Autres**	11(26,2)	1,5	0,5-4,6	0,52	-	-	-
Répartition des cas dans les villes de résidence (n= 2809)							
**Porto - Novo**	9(21,4)	1					
**Cotonou**	13(31)	0,7	0,3-1,6	0,40	-	-	-
**Parakou**	3(7,1)	0,1	0,0-0,5	**0,003**	-	-	-
**Natitingou**	17(40,5)	0,7	0,3-1,6	0,42	-	-	-
Antécédents							
Diabétique (n= 2809)							
**Oui**	2(4,8)	1,6	0,4-6,9	0,50	-	-	-
**Non**	40(95,2)	1			-	-	-
ATCD d´alcoolisme (n=2809)							
**Oui**	24(57,1)	8,5	4,5-15,7	**<0,0001**	6,3	3,3-12,1	**<0,0001**
**Non**	18(42,9)	1			1		
ATCD d´Hépatite B (n=2809)							
**Oui**	2(4,8)	0,7	0,2-2,8	0,58	-	-	-
**Non**	40(95,2)	1			-	-	-
(*): Données manquantes							

## Discussion

Cette étude avait permis d´estimer la séroprévalence de l´Hépatite C au Bénin et d´identifier les facteurs associés à cette infection. La prévalence globale de l´hépatite C dans notre série était de 1,5%. Cette prévalence est proche de la prévalence mondiale objectivée par l´OMS en 2015 (1%) [2]. Ce résultat corrobore ceux de Petruziello *et al*. [11] et d´Aboubacar *et al*. [12] qui avaient objectivé au Bénin respectivement une prévalence de 1,6% en 2016 et de 1,2% en 2020. Petruzziello *et al*. avaient fait une revue de la littérature de 2000 à 2015, alors qu´Aboubacar *et al*. avaient réalisé leur étude dans une population de gestantes dans plusieurs maternités de référence de Cotonou. Par contre, cette prévalence est largement en dessous de celles trouvées dans certaines études antérieures réalisées au Bénin [5-7]. Cette différence pourrait être en rapport avec le type d´échantillonnage et la population d´étude. De cette prévalence, il ressort que le Bénin est un pays de faible endémicité par rapport au VHC au même titre que le Nigéria avec une prévalence de 0,7% en 2019 [13]. Par contre, des prévalences élevées avaient été notées dans certains pays de l´Afrique comme le Cameroun où elle est de 2,55% en milieu urbain [14], 1,2 à 2,5% au Burkina Faso [15], et 6,7% au Gabon [16]. Ainsi, la prévalence de l´infection par le VHC varie un peu d´un pays à l´autre. Même au sein du pays, la prévalence varie d´une région à l´autre [17]; c´est ce que montrait par ailleurs notre travail où la ville de Parakou semblait moins touchée par le VHC que les autres. Ces variations sont en partie liées à la prépondérance variable des facteurs de risque de transmission selon les régions et les pays. Même si les modes de contamination ne sont pas bien compris en Afrique subsaharienne, il est retenu parmi les causes possibles, une sécurité transfusionnelle précaire ; les pratiques traditionnelles (scarification, rasage, tatouage et circoncision) seraient des facteurs de promotion de transmission du VHC en zone rurale et l´insuffisance voire l´absence de matériel et dispositifs médicaux à usage unique [14]. L´hypothèse d´une contamination massive des populations d´Afrique Centrale dans la zone endémique de la maladie de Sommeil (*Trypanosomiase africaine*) pourrait être une explication plausible de la forte prévalence de VHC observée dans certains pays de l´Afrique Centrale (Gabon) [14] en plus de la pratique traditionnelle commune en Afrique, de la précarité de sécurité transfusionnelle et l´insuffisance de matériel et dispositifs médicaux à usage unique [14].

Les facteurs associés à l´infection au virus de l´hépatite C (VHC) retrouvés dans notre étude étaient l´âge et l´alcoolisme. La tranche d´âge de 60 ans et plus était fortement associée à l´infection au VHC; les enfants sont très rarement concernés par le VHC. Les sujets de 60 ans et plus peuvent être considérés véritablement comme des personnes à risque du VHC dans notre pays et les interventions de dépistage devraient les cibler parmi les groupes prioritaires. Il avait été rapporté que la prévalence d´anticorps anti VHC, augmentait avec l´âge [17]. De plus la transfusion sanguine, les interventions chirurgicales, l´hémodialyse ou l´accouchement difficile, de même que certaines injections (défaut d´asepsie au soin, la réutilisation des aiguilles) avant 1992 étaient associés à un risque d´infection virale C du fait de la connaissance limitée sur le virus et son mode de transmission à l´époque [17, 18]. Quant à l´alcoolisme, il avait été incriminé pour accélérer l´évolution de l´infection au VHC vers la chronicité et la cirrhose [17]. On pourrait aussi penser que pendant l´éthylisme aigu, l´individu pourrait se blesser avec n´importe quel objet sans s´en rendre compte. Contrairement à l'antécédent de transfusion, la notion de scarification, de tatouage et de partage de matériels d´injection des drogues n'avaient pas été systématiquement recherchée chez les enquêtés. Oshun *et al*. au Lagos (Nigeria) en 2019 n´ont pas objectivé de facteurs ci-dessus énumérés comme étant associés à l´infection par le VHC, ni non plus la corrélation entre l´âge, l´alcoolisme et l´hépatite C [13].

De plus certaines études avaient rapporté l´implication de l´infection au VHC dans la genèse de diabète de type 2 [12, 17]. Cette corrélation infection VHC et diabète type 2 n´avait pas été retrouvée dans notre étude et pourrait s´expliquer d´une part par la faible participation de diabétique à notre étude. Une étude de prévalence de l´infection de VHC sur une plus grande population incluant des diabétiques ou une étude cas - témoins permettrait d´élucider cette corrélation [19]. Cette étude présente comme limite d´avoir porté sur des volontaires, et n´a pas couvert l´ensemble du territoire national béninois. Les données ne peuvent donc pas être généralisées à la population béninoise. En plus, les résultats positifs au test rapide n´ont pas été systématiquement confirmés par un test ELISA (Ezyme Linked Immuno-Sorbent-Assay), ni par PCR et donc la prévalence trouvée aurait pu être une surestimation en raison de possibles faux positifs ou de patients guéris du VHC. Toutefois, ce travail a le mérite de fournir des données préliminaires sur l´importance de l´infection par le VHC au Bénin, en attendant une étude bien menée de prévalence à l´échelle nationale.

## Conclusion

La séroprévalence des anticorps anti-VHC est en moyenne de 1,5% dans les quatre plus grandes villes du Bénin. L´infection par le VHC semblerait toucher volontiers les sujets âgés (de 60 ans et plus) et ceux ayant un antécédent d´alcoolisme chronique. Toutefois, l´étude ayant porté sur des volontaires au dépistage dans quelques villes du Bénin, une étude d´envergure nationale s´avère indispensable pour connaître la vraie prévalence du VHC au Bénin et en rechercher les réels facteurs de risque.

### Etat des connaissances sur le sujet

- L´Afrique subsaharienne, y compris au Bénin, est considérée comme une zone de moyenne endémicité pour l´infection par le VHC;
- Les principaux facteurs de risque de l´infection au virus de l´hépatite C sont la transfusion sanguine, la scarification, les tatouages, partage de seringue d´injection.

### Contribution de notre étude à la connaissance

- La séroprévalence des anticorps anti-VHC est de 1,5% dans les quatre plus grandes villes du Bénin;
- Les facteurs associés à cette infection sont : la tranche d´âge de 60 ans et plus, l´alcoolisme.

## Remerciements

Au groupe Lefalco de Cotonou, Bénin et au Laboratoire GENIX Pharma du Pakistan pour le financement et l´appui technique à la réalisation de la campagne du dépistage de l´hépatite C.
